# Research on Flocculant Selection for Classified Fine Tailings Based on Micro-Characterization of Floc Structure Characteristics

**DOI:** 10.3390/ma15072460

**Published:** 2022-03-27

**Authors:** Yuye Tan, Xiang Meng, Zhiwei Jiang, Chongchong Han, Mochuan Guo

**Affiliations:** 1 Key Laboratory of the Ministry of Education for High Efficient Mining and Safety in Mental Mines, Beijing 100083, China; tanyuye@ustb.edu.cn (Y.T.); 41827274@xs.ustb.edu.cn (Z.J.); g20198065@xs.ustb.edu.cn (C.H.); g20208008@xs.ustb.edu.cn (M.G.); 2 School of Civil and Resource Engineering, University of Science and Technology Beijing, Beijing 100083, China

**Keywords:** classified fine tailings, flocculation and sedimentation, nuclear magnetic resonance, SEM image, gray value

## Abstract

The rapid settlement of tailings is an important technical guarantee for the continuous production of downhole filling. The selection of a reasonable flocculant is essential for accelerating the settlement speed of classified fine tailings. The present paper conducts indoor static sedimentation experiments, NMR observation, electron microscope scanning, and other methods to analyze the porosity and pore-size distribution characteristics of floc solution for classified fine tailing under four flocculants, namely, ZYZ, ZYD, JYC-1, and JYC-2. The dimension, spatial distribution characteristics, particle size characteristics, and morphological characteristics of the scanning electron microscope images of floc were studied. Results show that the unit consumption of flocculant at 30 g/t is the critical value for increasing the flocculation and sedimentation effect of the classified fine tailings solution. The highest distribution percentage of small-sized classified fine tailings and the lowest average pore size was observed under the ZYZ-type flocculant. This flocculant also obtained the lowest porosity, largest average floc size, largest area occupied by the floc, lowest pore percentage, and the densest floc structure. Thus, this flocculant showed the best flocculation effect. A negative correlation was observed between the equivalent diameter of floc with varying settlement heights. The dimension of floc increased with the decrease in bed settlement height, and the overall structure of the floc gradually transitioned from loose to dense from top to bottom. The present paper characterizes the microscopic morphology and spatial structure characteristics of floc under different flocculants from a microscopic point of view. The present paper also provides a scientific basis for the selection of the optimal flocculant.

## 1. Introduction

Filling an underground mining area can effectively reduce surface tailing volumes, which is a priority in the green development of mines. At present, most underground gold mines use classified tailings, which are usually classified coarse tailings, to fill empty underground areas while fine tailings are discharged to tailing depots. Fine tailing particles have small particle sizes and large specific surface area. These particles can easily affect the stability and impermeability of tailings in a dam, which may lead to the formation of hazardous dams [1]. With the progress of solid waste utilization technology, coarse-grained tailings can be considered as a mineral in different applications [2], such as building materials and roadbed construction [3]. This development is a key measure to build tailless mines, reuse the resources of coarse-grained tailings after grading, fill underground empty areas with classified fine tailings, and achieve 100% comprehensive utilization of tailing resources [4,5,6,7]. In many metal mines, the tailings’ thickening and dewatering process is the premise of the paste filling process. In order to increase the settlement rate of tailings, it is convenient and operable to add a flocculation agent to the tailings slurry. Now flocculation settlement technology has been widely used in the thickening and dewatering of mine tailings [8,9,10]. However, current studies on flocculation settlement characteristic are mainly based on unclassified tailings. It is generally known that classified fine tailings have a lower settlement rate, which seriously affects the efficiency and continuity of underground filling; thus, a flocculation agent should be added to speed up settlement.

Scholars have conducted a large number of indoor static experiments to examine the factors that affect tailing settlement. Du Jiafa [11], Zhang Meidao [12], Li Jinxin [13], and Shi Caixing [14] obtained the best flocculation agent consumption, and other parameters, by conducting a static flocculation settlement experiment on full tailings. In their flocculation settlement experiment, Gao Zhiyong [15] optimized tailing concentration, flocculant solution, and dosage. Other scholars also studied the factors that affect tailing settlement by establishing mathematical models and equations. Wu Aixiang [16,17] obtained the best flocculation conditions and optimized the settling and flocculation parameters of tailings mortar based on super flocculation theory and the response surface method. Wen Zhenjiang [18] studied the effect of multi-factor interaction on the settlement of whole tailings’ flocculation by establishing a regression model of the response surface. The evolution of the size of whole tailings under different flocculation conditions was explored by measuring the string length of the floc [19]. However, few scholars characterized the micro-form and size of floc tailings from a microscopic perspective. Hou Hezi [20] observed the flocculation samples of tailings through environmental scanning electron microscopy and studied the rate of flocculation settlement of tailings, which can be divided into five stages. Yang Zilong [21] used magnetic resonance imaging to explore the distribution and porosity characteristics of floc apertures at different heights. The present paper focuses on exploring the factors that affect tailing flocculation through indoor static settlement experiments. The subjects are generally whole tailings or classified coarse tailings. Few studies with limited results were conducted on the subtle features of floc in classified fine tailings.

The present paper proposes to obtain samples of the best floc solution for classified fine tailings under single-consumption flocculant by means of an indoor static settlement experiment, including nuclear magnetic resonance observation and microscope scanning. The present paper also analyzes the porosity and aperture distribution characteristics of flocculation solution for tailings. The structural characteristics of floc under different flocculants are characterized at a microscopic perspective by analyzing the flocculation size, spatial distribution characteristics, particle size characteristics, and morphological characteristics of scanning electron microscope (SEM) images of floc. The results provide a scientific basis for selecting optimal flocculants.

## 2. Experimental Materials and Programs

### 2.1. Experimental Materials

#### 2.1.1. Classified Fine Tailings

**Figure 1:**
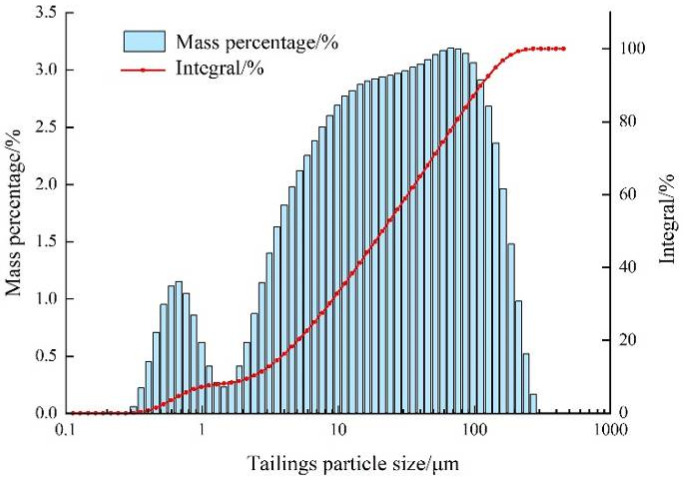
Particle size distribution diagram of classified fine tailings.

The experimental tailings used in the present paper were classified fine tailings from a gold mine. Figure 1 and Table 1 show the particle size distribution and physical properties of the samples. The analysis shows that the particle size of tailings is mainly distributed between 5 μm and 160 μm, and about 98% of the tailings particles are less than 200 μm. The weighted average of the particle size is 43.88 μm and the median particle size is 24.10 μm. The main chemical composition of classified fine tailings is shown in Table 2.

#### 2.1.2. Flocculants

Four anion-type flocculants were selected according to the types of flocculants commonly used in the settlement of gold tailings. The flocculants are ZYZ, ZYD, JYC-1, and JYC-2, and their parameters are shown in Table 3.

### 2.2. Experimental Programs and Equipment

#### 2.2.1. Static Flocculation Settlement Experiment

The optimal single-consumption flocculation agent was first determined by a static flocculation settlement experiment to obtain quality tailings flocculation. The mass concentration of tailings solution and the unit consumption of flocculant are the two most important factors affecting tailings settlement. According to statistics, for the tailings of some mines, the optimum tailings solution concentration ranges from 5% to 25% [22]. In order to simplify the experimental scheme and achieve the experimental purpose, a single consumption of flocculant was used as the only variable in this experiment. In this paper, a single consumption of flocculant was measured at 0 g/t, 10 g/t, 20 g/t, 30 g/t, and 40 g/t. The concentration of tailings solution was 20%, and the concentration of flocculant solution was 0.3%. Table 4 shows the experimental grouping. The height of the mud layer at different times during the experiment was recorded.

#### 2.2.2. Nuclear Magnetic Resonance Observation Experiment

**Figure 2:**
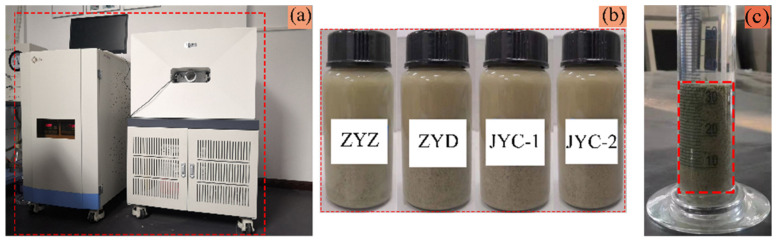
Nuclear magnetic resonance observation experiment: (**a**) MRI analyzer; (**b**) samples of floc solution; (**c**) floc extraction location.

Four classified fine tailings solutions with concentration of 20% were configured. ZYZ, ZYD, JYC-1, and JYC-2 flocculants were added to the solutions using the best single-consumption flocculant and flocculant solution at 0.3% concentration. After stirring, the solutions were transferred to different acrylic bottles. The porosity and aperture distribution of the floc solutions of classified fine tailings were observed by a magnetic resonance imaging analyzer, as shown in Figure 2.

A magnetic resonance imaging analyzer was used, with 23 MHz resonance frequency, magnet temperature ranging from 25 °C to 35 °C, and a Spiral-Sprite imaging sequence.

#### 2.2.3. Scanning Electron Microscopy Experiment

**Figure 3:**
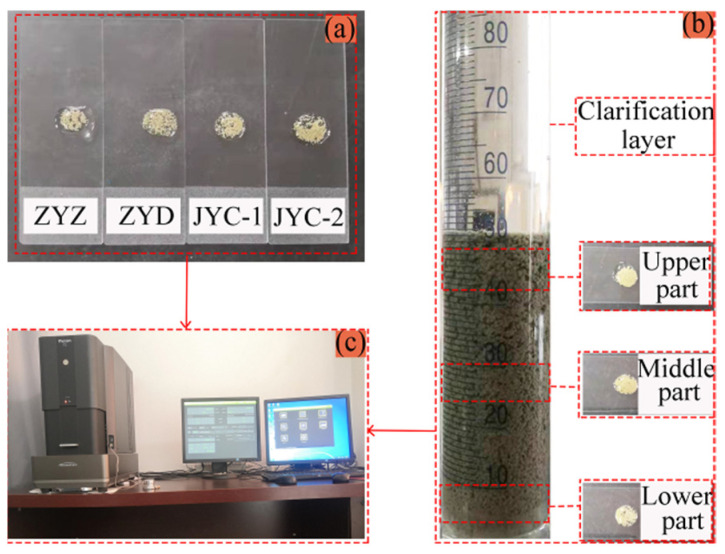
Electron microscope scanning experiment: (**a**) floc samples under four flocculants; (**b**) sampling positions at different height in a volumetric flask; (**c**) Phenom XL desktop SEM.

First, the four samples of floc solutions of classified fine tailings obtained from the MRI observation experiment were converted into microscope scanning samples, as shown in Figure 3a. The production process was as follows. The floc solution of tailings was placed on a slide and hung naturally for 24 h until dry. The solution was fixed with K-300 shadowless glue. The gold spray process was conducted before scanning observation. The SEM images of the floc tailings were magnified 5000 times by microscope scanning.

The optimal flocculation agent was selected to configure the floc solution of classified fine tailings. The tailings solution had a concentration of 20%, and the concentration of the flocculant solution was 0.3%. The best flocculation agent was consumed in a static flocculation settlement experiment. Due to the increasing height of the tail sand bed layer formed by the deposition of floc to the bottom, the pressure of the bed layer gradually increased from top to bottom, and the gap between floc decreased continuously [23]. Therefore, floc in the upper, middle, and lower parts of the bed layer were taken separately. The microscope scanning samples were selected, as shown in Figure 3b, to obtain the best SEM images of floc with different settling heights.

Phenom XL benchtop SEM is used, with high brightness CeB6 filament, resolution of less than 10 nm, and operating voltage of 15 kV, as shown in Figure 3c.

## 3. Experimental Results and Analysis

### 3.1. Analysis of Best Single-Consumption Flocculant

**Figure 4:**
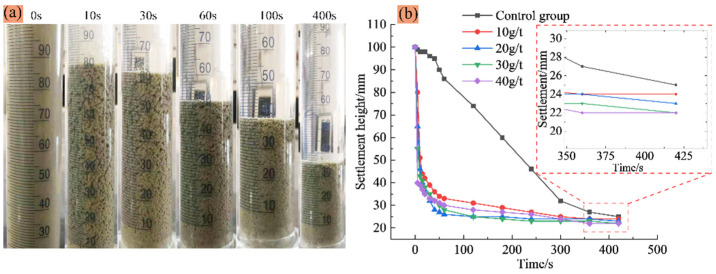
Settlement process of floc (**a**) and change curve of height with time under different flocculant consumption (**b**).

Figure 4 shows the settlement process of floc and the variation curve of the single-consumption settlement height of flocculant ZYZ over time obtained through a static flocculation settlement experiment. Analysis shows that the addition of flocculant significantly accelerated the pre-settling speed of classified fine tailings. Four groups of classified fine tailings of floc solution occurred at 0 s to 60 s rapid settlement process. The five groups of experiments were completed at about 400 s. The final settling heights were 24 mm, 23 mm, 22 mm, 22 mm, and 25 mm, respectively. The lower the final settling height, the more compact the floc were, which resulted in an ideal flocculation settlement effect. The final settlement height decreased with the increase in flocculant consumption at a certain range of flocculant consumption. The final settling height did not change when the single consumption of flocculant increased from 30 g/t to 40 g/t. The result shows that flocculant consumption of 30 g/t is the threshold for enhancing the flocculation settling effect, which can be used as the best value for single-consumption flocculant.

### 3.2. Analysis of Aperture Distribution Characteristics and Porosity of Floc Solution

**Figure 5:**
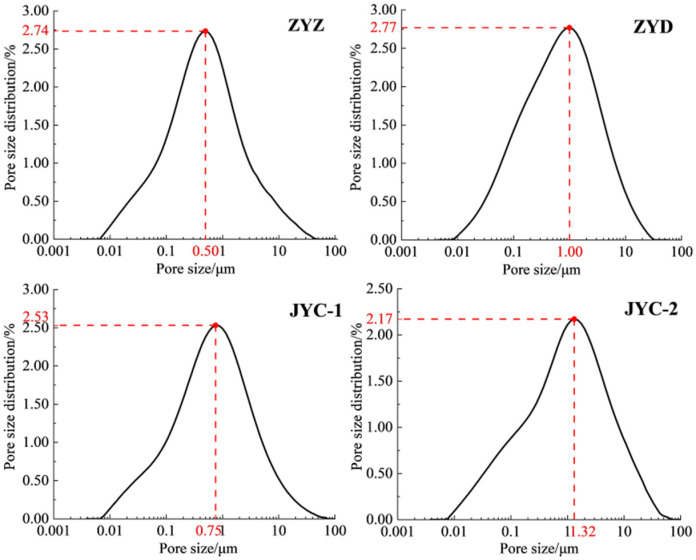
Curve of pore size distribution percentage of floc under different flocculants.

Figure 5 shows the aperture size that corresponds to the maximum percentage of aperture distribution of floc solutions under the four flocculants, which is sorted in the following order: ZYZ < JYC-1 < ZYD < JYC-2. The maximum aperture ratio of floc solution for the classified fine tailings under ZYZ and JYC-1 is 2.74% and 2.53%, respectively. The corresponding aperture sizes are 0.50 μm and 0.75 μm, respectively, which are both less than 1.00 μm. The maximum aperture ratio of floc solution of the classified fine tailings under ZYD and JYC-2 is 2.77% and 2.17%, respectively. The corresponding aperture sizes are 1.00 μm and 1.32 μm, which are higher or equal to 1.00 μm.

**Figure 6:**
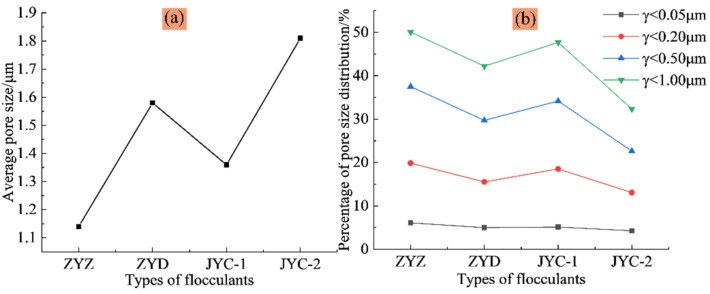
Pore size distribution characteristics of floc solution under different flocculants: (**a**) average pore size; (**b**) pore size distribution percentage.

As shown in Figure 6a, the overall average aperture of the solution and the distribution percentage of the four intervals of solution aperture sizes of 0 to 0.05 μm, 0 to 0.20 μm, 0 to 0.50 μm, and 0 to 1.00 μm are counted to determine the distribution of small and medium-sized apertures in the floc solution of classified fine tailings. The average aperture size of floc solution of classified fine tailings is sorted as follows: ZYZ < JYC-1 < ZYD < JYC-2. Figure 6b shows the percentage of aperture distribution, which is sorted as: ZYZ > JYC-1 > ZYD > JYC-2. The highest distribution percentage of small size aperture and the lowest distribution of average aperture of classified fine tailings solution were observed under the ZYZ-type flocculation solution. This finding indicates that the smallest spacing between floc was found under this flocculant, which demonstrated the densest floc structure.

In terms of porosity, ZYZ, ZYD, JYC-1, and JYC-2 flocculation solution have porosity percentages of 67.23%, 70.72%, 68.35%, and 78.40%, respectively. The lowest porosity was observed under the ZYZ flocculant, which indicates that the largest average size of floc was observed in this flocculant.

### 3.3. Analysis of Floc Size and Spatial Distribution Characteristics

#### 3.3.1. Analysis of Floc Size Characteristics Based on Gray Scale Measurement

**Figure 7:**
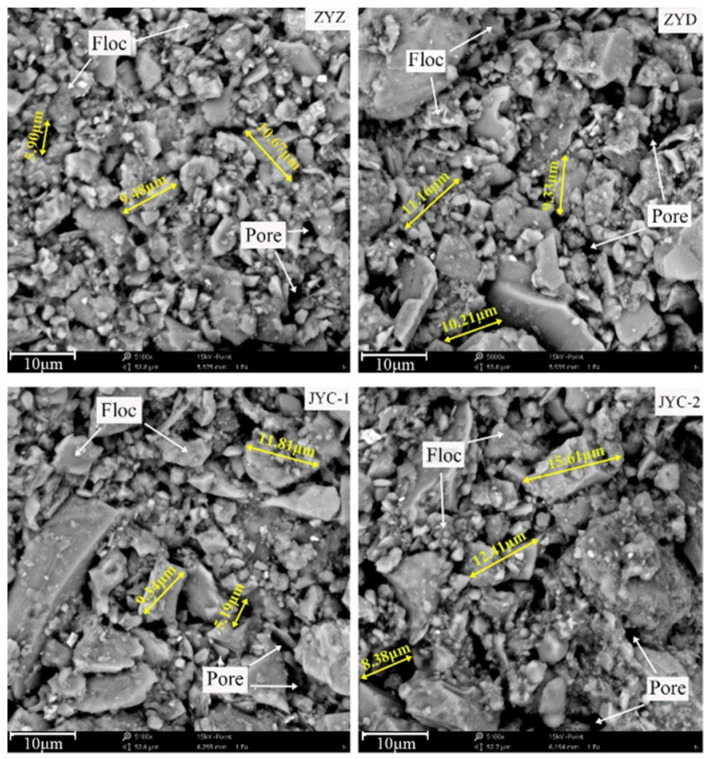
SEM images of floc under different flocculants.

According to the grayscale measurement principle, the grayscale value of floc in SEM images is high, and the grayscale value of pores is low, which means that grayscale value can be used to distinguish between floc tailings and pores [24]. Figure 7 shows the SEM images of floc samples magnified about 5000 times. The floc is gray and the pores are black. The contours of floc and the pores between floc are clearly visible.

**Figure 8:**
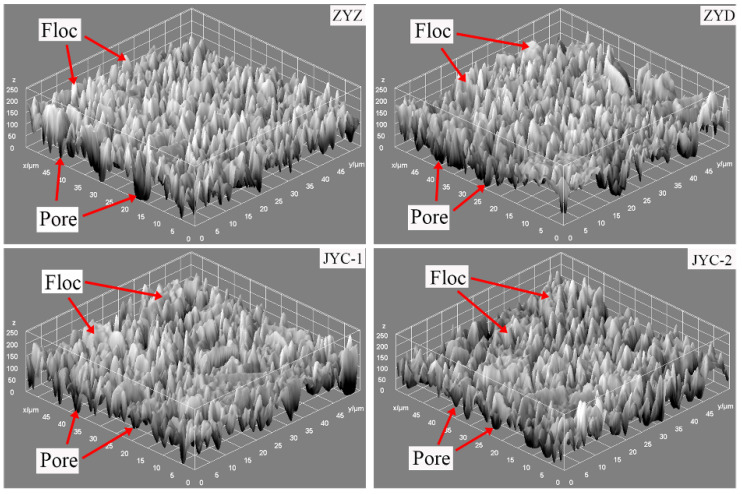
Grayscale measurement images of floc.

In order to preliminarily judge the relative proportion of floc and pores, the overall grayscale measurement of the floc was carried out and grayscale three-dimensional images were obtained, as shown in Figure 8. It can be seen from the measurement results that the average gray values of floc under the four flocculants ZYZ, ZYD, JYC-1, and JYC-2 are 122, 116, 119, and 107, respectively. The larger the average gray value is, the wider the distribution range of floc is, and the less the number of pores. Based on this, it is preliminarily determined that the percentage of pore distribution in tailings floc is sorted as follows: ZYZ < JYC-1 < ZYD < JYC-2; then the overall size of floc is sorted as: ZYZ > JYC-1 > ZYD > JYC-2.

#### 3.3.2. Analysis of the Spatial Distribution Characteristics of Flocs Based on Binarized Images

**Figure 9:**
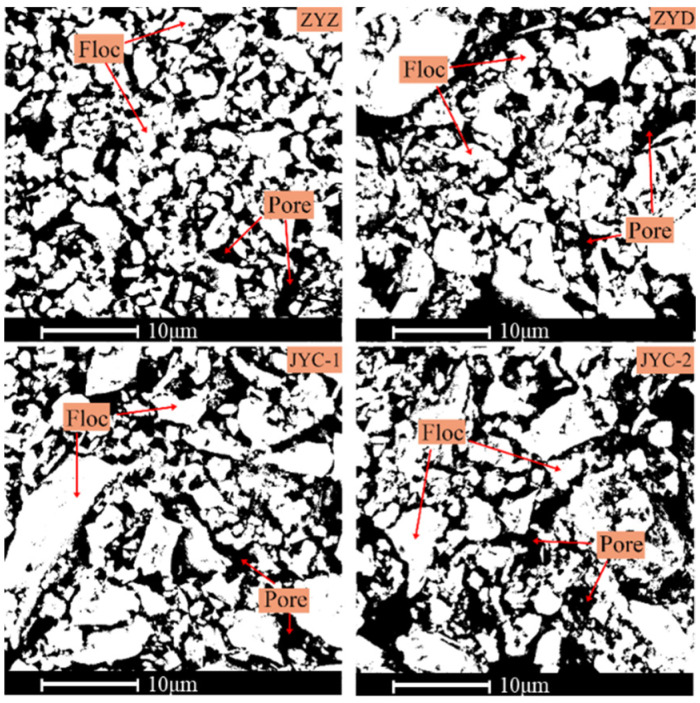
Binarized images of floc under different flocculants.

A reasonable segmentation threshold is selected to binarize the SEM images in Figure 7 to obtain the proportion of floc in SEM images. The binary images of floc are shown in Figure 9, wherein the white area indicates the floc and the black area is the pores between floc.

The grayscale values of processed SEM image are only 0 and 256. The following models are established based on mathematical statistics [25,26]. If *X* ~ *N*(0, 1) and *P*(*X* = 0) = *p* and *P*(*p*_1_ < *p* < *p*_2_) ≈ 1 − α, then an approximate confidence interval of *X* is (*p*_1_, *p*_2_) when the confidence level of *p* is 1 − α. Figure 8 shows grayscale values of only 0 and 256, which obey the 0–1 distribution model in mathematical statistics. Thus, the overall grayscale value of the images can be estimated by the grayscale value of the selected sample points. As shown in Table 5, a number of sample points are selected in the images, and the numbers of points with grayscale values of 0 or 255 are counted. The confidence intervals of points with grayscale value of 255 are calculated using Equations (1)–(3). The confidence level is 0.95, which represents the percentage range of floc in the binary images.

(1) $$p_{1}=\frac{1}{2a}(-b-\sqrt{b^{2}-4ac}),p_{2}=\frac{1}{2a}(-b+\sqrt{b^{2}-4ac})$$

(2) $$a=n+z^{2}{}_{\frac{\alpha }{2}},b=-(2n\bar{X}+z^{2}{}_{\frac{\alpha }{2}}),c=n{\bar{X}}^{2}$$

(3) $$P=(p_{1}<p<p_{2})\approx 1-\alpha$$

Table 6 shows the floc percentage range of ZYZ, ZYD, JYC-1, JYC-2 at (58.7%, 64.7%), (55.5%, 61.5%), (56.6%, 62.7%), and (52.9%, 59.0%), respectively, when the confidence level is 0.95. The result is ZYZ > JYC-1 > ZYD > JYC-2 when the lower and upper confidence interval values are sorted separately. Results show that the grayscale value of the tail sand flocculation group under ZYZ flocculant is the highest number at 255 points. This result indicates the largest area occupied by floc, which has the lowest pore percentage content and the tightest overall flocculation structure. The results of the analysis further confirm the results of previous analysis that the ZYZ flocculant is suitable for the classified fine tailings of the optimal flocculant model.

#### 3.3.3. Fractal Characteristics of Floc Structure of Classified Fine Tailings

The fractal theory is a discipline that studies a kind of irregular and chaotic object with similarity of its part and whole. Fractal dimension constitutes the main tool to test for fractal patterns in Euclidean contexts. The (lower/upper) box dimension for any subset *F* ⊆ Rd is given by the following (lower/upper) limit:

(4) $$\dim_{B}(F)=\underset{\delta \to 0}{\lim}\frac{\log N_{\delta }(F)}{-\log\delta }$$

where *N_δ_* (*F*) is the number of *δ*-cubes that intersect *F* [27].

**Figure 10:**
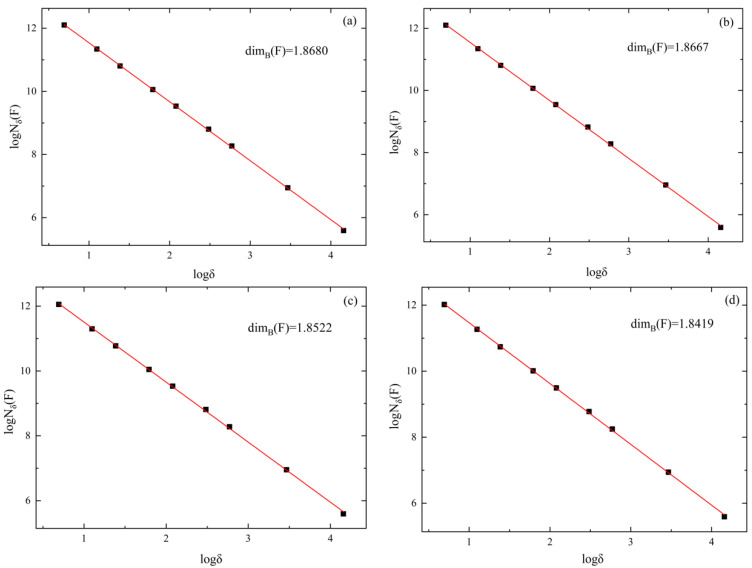
Fractal characteristic curve of floc structure: (**a**) ZYZ; (**b**) JYC-1; (**c**) ZYD; (**d**) JYC-2.

Classified fine tailings floc is an irregular mass formed by the collision and combination of tailings particles and flocculants under the action of Brownian motion and turbulence. The formation process of floc shows that the floc has fractal characteristics, so the fractal dimension can be used as a quantitative control parameter and the flocculation effect of the flocculant can be compared by comparing the size of the fractal dimension. As shown in Figure 10, fractal dimensions of classified fine tailings under the four flocculants are 1.8680, 1.8667, 1.8522, and 1.8419, respectively. It can be seen that the fractal dimension of the floc structure under ZYZ flocculant is the largest, which means the floc is the most compact, the particle spacing inside the floc is the smallest, the difference between the floc density and the liquid density is the largest, and the settling velocity is the largest, so the flocculation effect most. According to the fractal theory, the order of the flocculation effect of the four flocculants on the classified fine tailings is ZYZ > JYC-1 > ZYD > JYC-2.

### 3.4. Analysis of the Morphology and Spatial Structure Characteristics of Floc

**Figure 11:**
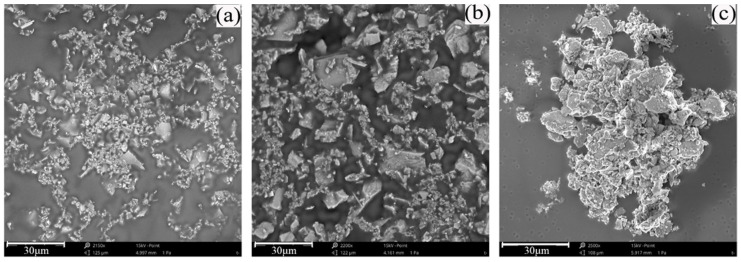
SEM images of floc with different settlement heights under ZYZ type flocculant: (**a**) upper part; (**b**) middle part; (**c**) lower part.

The optimal flocculant was selected to configure the floc solution of classified fine tailings. As shown in Figure 11, the upper, middle, and lower parts of the sedimentary mud layer (Figure 3) were taken to produce microscope scanning samples and obtain SEM images of floc at different settlement heights.

#### 3.4.1. Analysis of the Granularity and Morphological Characteristics of Floc

**Figure 12:**
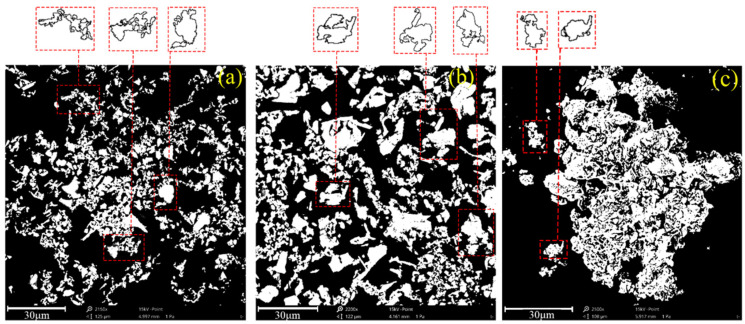
Particle size and morphological characteristics of floc with different settlement heights under ZYZ type flocculant: (**a**) upper part; (**b**) middle part; (**c**) lower part.

The SEM images of the floc at different settlement heights were binarized. To obtain the perfect image profile, edge detection was performed, and the boundary discrete points were connected to a closed curve [28,29,30,31]. The area of the closed floc tailings block is shown in Figure 12, and the white part of the figure is the floc. The profile of the floc is clear, and the particles at different settlement heights show distinct characteristics. The upper floc are more dispersed, the middle floc are larger in size, and the lower floc are denser.

**Figure 13:**
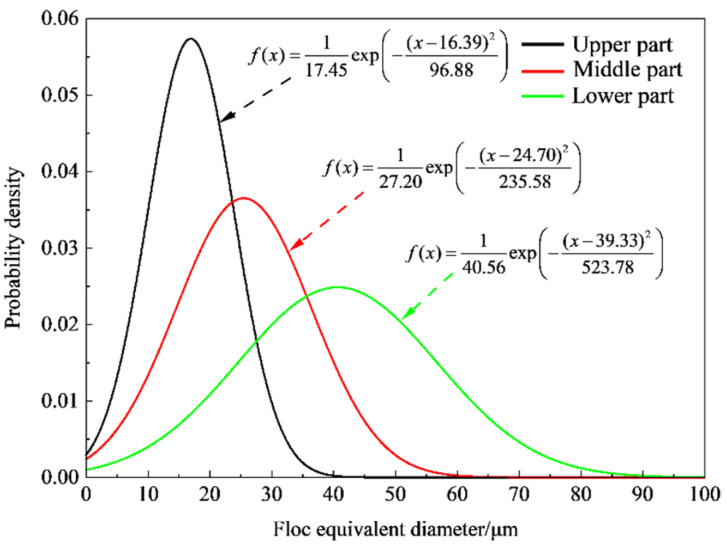
Distribution curve of equivalent diameters of floc at different heights under ZYZ type flocculant.

The equivalent diameter of the floc was obtained according to the closed area of the selected floc contour. The normal distribution curve of the tail-sand floc size was obtained through fitting, as shown in Figure 13. Small-size floc account for a large proportion in the upper part, which range from 5 to 30 μm. The size of the middle floc has increased and is concentrated between 10 and 40 μm. The lower large-size floc account for a large proportion and are concentrated at 20 to 60 μm. The average equivalent diameters of the upper, middle, and lower floc are 16.39 μm, 24.70 μm, and 39.33 μm, which indicate that the diameter of the floc is negatively correlated with settlement height.

#### 3.4.2. Analysis of the Spatial Structure Characteristics of Floc

**Figure 14:**
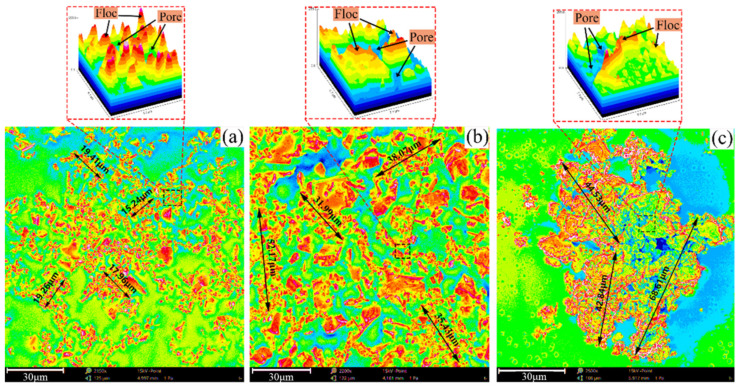
Spatial morphology of floc with different settlement heights under ZYZ type flocculant: (**a**) upper part; (**b**) middle part; (**c**) lower part.

The SEM images are colored, as shown in Figure 14, to analyze the spatial and morphological characteristic of floc at different settling heights. The sizes of the floc in the upper sample are small, which range from 15.24 μm to 19.41 μm. The overall structure of floc is loose. The size of the middle floc increased, some of which are between 31.99 μm and 52.17 μm. The mass structure of the floc is denser than that of the upper floc. The overall size of the lower floc is the largest, and range between 42.84 μm and 68.61 μm. Floc structure is densest in the lower floc.

The size of the floc increases with the decrease in settlement height of the bed layer because a gap exists between the floc after the end of the flocculation phase. Thus, floc could continue to settle under gravity, and gap moisture is squeezed out because of floc pressure. The floc that are originally separated by gap water can then touch each other, which results in a corresponding reduction in the pores of the floc and an increase in floc size. The lower the settlement height of the mud layer, the stronger the pressure on the gap moisture, and the higher the amount of discharge. Therefore, the size of floc increases from top to bottom, and the overall structure of the floc gradually transitions from loose to dense.

## 4. Conclusions

The porosity and aperture distribution characteristics of floc solution of the classified fine tailings were analyzed through indoor static subsidence, MRI observation, and micro-electric scanning. The microstructure of floc were characterized by analyzing the size and spatial distribution characteristics of floc under different flocculants and the granularity and spatial morphological characteristics of floc at different heights of mud layers. The analysis provides a scientific basis for the selection of optimal flocculation agent from the microscopic angle. The main conclusions are as follows.

(1) The addition of flocculant significantly accelerates the pre-settling speed of classified fine tailings. The tailings flocculation solution basically occurs in the rapid settlement process from 0 s to 60 s. The final settlement height decreases with the increase in flocculant consumption at a certain range of flocculant consumption. Flocculant consumption of 30 g/t is the critical value for enhancing the effect of flocculation settling. This value is selected as the best single-consumption value of flocculant.

(2) The analysis of MRI observations showed that the largest percentage of small aperture distribution, the smallest average aperture, and the lowest porosity of floc solution were observed under the ZYZ flocculant. This result shows that floc spacing in the tailings solution was smallest under the ZYZ flocculant. The largest average size of floc and the densest floc structure were also found under the ZYZ flocculant.

(3) The binary analysis of SEM image shows the largest area of classified floc was found under the ZYZ flocculant. The lowest pore percentage content, the largest average size of floc, the densest floc structure, and the optimal flocculation effect were also observed under the ZYZ flocculant.

(4) The particle granularity and spatial morphology characteristics of floc were analyzed at different settling heights under the ZYZ flocculant. Results show that the average equivalent diameters of the sedimentary mud layer of the upper, middle, and lower floc are 16.39 µm, 24.70 µm, and 39.33 µm, respectively. These results indicate that the equivalent diameter of floc is negatively correlated with settlement height. The size of the floc increases with the decrease in settlement height of the mud layer. The overall structure of the flocculation from top to bottom gradually transitions from loose to dense.

## Figures and Tables

**Table 1:** Physical properties of classified fine tailings.

Material Name	Specific Gravity	Loose Density/(g·cm^−1^)	Average Particle Size/μm	Porosity/%
Classified fine tailings	2.55	0.86	13.20	57.36

**Table 2:** Main chemical composition of classified fine tailings.

Chemical Composition	SiO_2_	Al_2_O_3_	CaO	Fe_2_O_3_	K_2_O	MgO	Na_2_O	TiO_2_
Percentage/%	61.754	12.689	9.182	5.953	4.437	3.305	1.244	0.650

**Table 3:** Parameters of different types of flocculants.

Types of Flocculants	ZYZ	ZYD	JYC-1	JYC-2
Molecular weight	16–18 million	16–18 million	16 million	18 million
State	White solid particles			

**Table 4:** Grouping table of static flocculation and settlement experiment.

Experiment Group Number	1	2	3	4	5 (Control Group)
Unit consumption of flocculant/(g·t^−1^)	10	20	30	40	0

**Table 5:** Numer of counted points with grayscale value of 0 or 255.

Flocculants	Number of Sample Points	Number of Points with Grayscale Value of 255	Number of Points with Grayscale Value of 0
ZYZ	1020	630	390
ZYD	1022	598	424
JYC-1	1022	610	412
JYC-2	1022	572	450

**Table 6:** Confidence interval of floc percentage content.

Flocculant	Number of Samples	Number of Points (Gray Value Is 255)	Number of Points (Gray Value Is 0)	Confidence Level (α)	Confidence Interval
ZYZ	1020	630	390	0.95	(58.7%, 64.7%)
ZYD	1022	598	424	0.95	(55.5%, 61.5%)
JYC-1	1022	610	412	0.95	(56.6%, 62.6%)
JYC-2	1022	572	450	0.95	(52.9%, 59.0%)
